# Establishing an auxin-inducible GFP nanobody-based acute protein knockdown system to mimic hypomorphic mutations during early medaka embryogenesis

**DOI:** 10.1242/bio.062081

**Published:** 2025-11-07

**Authors:** Kaisa Pakari, Sevinç Jakab, Johanna Rasch, Encarnación Sánchez Salvador, Beate Wittbrodt, Christian Thiel, Joachim Wittbrodt, Thomas Thumberger

**Affiliations:** ^1^ COS, Centre for Organismal Studies Heidelberg, Department of Developmental Biology/Physiology, Heidelberg University, 69120 Heidelberg, Germany; ^2^ HBIGS, Heidelberg Biosciences International Graduate School, Heidelberg University, 69120 Heidelberg, Germany; ^3^ Center for Child and Adolescent Medicine, Department Pediatrics I, Heidelberg University, 69120 Heidelberg, Germany

**Keywords:** Degron, Medaka, Endogenous GFP-tagging, CRISPR/Cas9, PMM2-CDG, Congenital disorders of glycosylation

## Abstract

Creating hypomorphic mutations is crucial to study gene function *in vivo*, especially when null mutations result in (embryonic) lethality. This applies to enzymes involved in glycosylation that, when mutated in human patients, cause the disease congenital disorders of glycosylation (CDG). In order to resemble patient condition, it would be ideal to acutely modulate the proteins in question to directly interfere with protein levels of such essential enzymes. These methods offer to establish pathogenic enzyme levels resembling net enzyme activity reported in patients suffering from CDG, with phosphomannomutase 2-CDG (PMM2-CDG) as the most common form.

We established an auxin-inducible acute protein knockdown system for the use in the teleost fish medaka (*Oryzias latipes*) by combining an improved degron (AID2) technology with an mAID-nanobody targeting endogenously GFP-tagged Pmm2 protein. We generated a fishline expressing a functional Pmm2-GFP fusion protein, by single copy integration of *GFP* into the *pmm2* locus. Upon induction, the degron system efficiently reduced Pmm2-GFP levels and enzyme activity, recapitulating the activity level of the hypomorphic mutations associated with PMM2-CDG in patients. This broadly applicable approach enables the investigation of CDG disease mechanisms during early embryonic development through reduction of protein abundance, mimicking hypomorphic mutations and thus substantially expanding the range of the genetic toolbox.

## Teaser

**Summary:** The combination of TIR1F74G and mAID-GFP-nanobody enables efficient acute knockdown of endogenously GFP-tagged proteins in medaka. This approach successfully reduced Pmm2 enzyme activity to pathogenic levels as seen in PMM2-CDG patients.

## INTRODUCTION

Multisystemic diseases are often caused by malfunctioning proteins, which result from genetic mutations that impact on enzyme activity. Animal models are crucial to understanding the molecular mechanisms underlying disease initiation and progression, and they offer a way to evaluate potential therapeutic treatments of such diseases. However, generating viable animal models by precisely recreating such mutations presents multiple challenges: targeting essential genes with the available genome editing toolkits causes unwanted insertion and deletions or coding sequence changes that are often lethal for the genome edited organism. This makes creating, identifying and breeding hypomorphic alleles nearly impossible. Further, stable genetic lines can only be maintained in a heterozygous state, i.e. due to Mendelian genetics, only a quarter of the offspring will be homozygous and may develop the (rare disease) phenotype.

An alternative and more direct approach is the manipulation of protein abundance, which allows us to mimic hypomorphic conditions in a controlled manner. This is especially important for studying rare human diseases such as congenital disorders of glycosylation (CDG). This rare group of complex metabolic disorders is caused by disrupted glycosylation, a critical post-translational modification process. CDG patients usually harbor hypomorphic mutations, which reduce enzyme activity and – compared to non-viable null mutations – cause less severe but often systemic symptoms. The most prevalent form of CDG is caused by mutations in the *Phosphomannomutase 2* (*PMM2*) gene (Ng et al., 2024) encoding the enzyme that converts mannose-6-phosphate to mannose-1-phosphate, the initial step for all endoplasmic reticulum (ER)-based glycosylation (*N*-glycosylation, *C*- and *O*-mannosylation, GPI anchor biosynthesis) (Sharma et al., 2014). Most of the reported PMM2-CDG patients have clearly reduced PMM2 enzyme activity (Pajusalu et al., 2024). Severe variants result in residual PMM2 enzyme activity below 10%, while the healthy heterozygous parents show over 50% activity (Westphal et al., 2001), suggesting pathogenicity below a threshold level of 50%. Various cell line models and studies, including patient-derived fibroblasts that carry hypomorphic PMM2 mutations, have helped to acquire a basic understanding of PMM2 activity on the cellular level. Available organismal models that are based on genetic PMM2 mutations either show a subset of patient phenotypes or suffer from early lethality (Chan et al., 2016; Cline et al., 2012; Himmelreich et al., 2015; Parkinson et al., 2016; Schneider et al., 2012; Thiel et al., 2006; Zhong and Lai, 2025).

In contrast, controlled reduction of PMM2 protein abundance would affect all treated specimens and result in reduced net enzyme activity, comparable to the hypomorphic conditions seen in patients. Progressive diseases like PMM2-CDG are characterized by an early onset of phenotypes which is challenging to study in mammals due to the intrauterine development of the embryo. We thus turned to the teleost fish medaka (*Oryzias latipes*), a well-suited model for studying human diseases and related gene functions (Doering et al., 2023; Gücüm et al., 2021; Hammouda et al., 2021), as early embryogenesis can be thoroughly examined due to the extra-uterine development and transparency of the embryos (Wittbrodt et al., 2002).

As an alternative to recreating patient-specific alleles, controlled reduction of the PMM2 protein abundance can recreate patient conditions. An efficient method for acute and inducible reduction of PMM2 activity at the organismal level, targeted protein degradation using degron systems would provide the most direct approach.

Degron systems allow fast degradation of proteins-of-interest via the ubiquitin-proteasome pathway in a highly specific, inducible and reversible manner. The targeted inactivation of proteins can be achieved by the auxin-inducible degron (AID) system, an E3 ubiquitin ligase complex, in which the plant F-box protein TIR1 (transport inhibitor response 1) dimerizes with AID-tagged proteins in the presence of the plant hormone auxin (Nishimura et al., 2009). This dimerization recruits the tagged proteins to the endogenous Skp1–Cul1–F-box (SCF) E3 ligase complex and results in ubiquitination and degradation of the tagged protein. A drawback of the canonical TIR1 protein is its basal activity in the absence of auxin (Natsume et al., 2016). Improved degron systems differ between TIR1 F-box variants employed (Nishimura et al., 2020; Yesbolatova et al., 2020). Another limiting factor for these approaches is the requirement of tagging the protein-of-interest with AID. To gain flexibility and foster targeted line generation with a visible selection readout, we used a deGradFP approach in which GFP-tagged proteins are recognized by a highly GFP-specific nanobody (vhhGFP4) (Caussinus et al., 2011; Caussinus and Affolter, 2016) fused to a minimized mAID (Daniel et al., 2018). Successful degradation is thus visible due to the loss of fluorescence linked to target protein depletion upon induction.

Thus, generation of a stable Pmm2-GFP fusion line in medaka not only allows for acute interference with a visual readout but additionally provides a systemic expression analysis of Pmm2 *in vivo*.

Here we combined a specificity-improved auxin-inducible degron system [TIR1(F74G)] with an mAID-nanobody targeting GFP-tagged protein approach for the use in medaka. We generated and functionally validated an endogenously tagged Pmm2-GFP medaka line. Furthermore, we used a visible readout to demonstrate the efficacy of the combinatorial degron system by interfering with Pmm2-GFP levels and biochemically confirmed the reduced net enzyme activity of Pmm2 following induction of the degron system. We established a flexible system to manipulate Pmm2 levels and activity in a specific and efficient manner, providing a valuable tool for further studies on the role of Pmm2 in development.

## RESULTS AND DISCUSSION

**Fig. 1.**
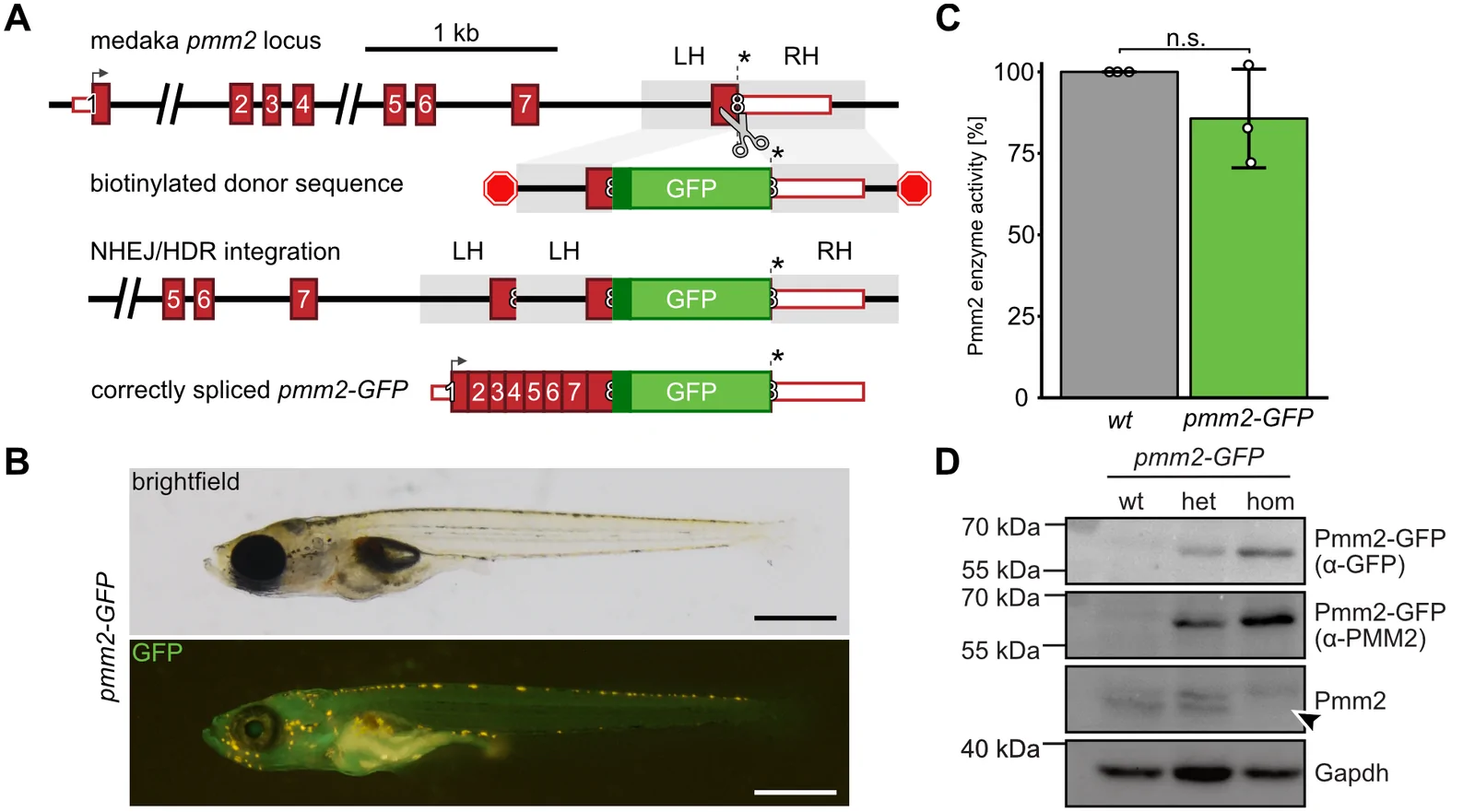
**Endogenous *GFP*-tagging of *pmm2* does not interfere with Pmm2 function in medaka.** (A) Schematic representation of the medaka *pmm2* gene locus. Endogenous GFP knock-in outline at the C-terminus of the *pmm2* coding sequence via CRISPR/Cas9 (scissors) and biotinylated (red stop signs) donor template [coding sequence, red boxes; untranslated region (UTR), white boxes]. Single copy integration by homology directed repair (HDR) with the right homology flank (RH) and a non-homologous end-joining (NHEJ) event of the left homology flank (LH). Transcript analysis revealed correctly spliced *pmm2-GFP* mRNA. (B) Representative hatchling homozygous for *pmm2-GFP* shows ubiquitous GFP expression and proper development. (C) Biochemical Pmm2 enzyme activity assay highlights matching levels of wild-type (wt) and GFP-tagged Pmm2. Results from three independent experiments with lysates of pooled embryos (*n*=25, 34 and 34). Mean±s.d. are shown, *t*-test, ns, *P*>0.05. (D) Western blot analysis of lysates of wild-type (wt), Pmm2-GFP heterozygous (het) and Pmm2-GFP homozygous (hom) hatchlings (*n*=9 pooled hatchlings per lysate for each genotype), reveal the presence of Pmm2-GFP as a stable fusion protein using Pmm2 and GFP-specific antibodies; see absence of Pmm2 band in hom hatchlings (arrowhead), Gapdh was used as loading control. Scale bars: 500 µm. *, stop-codon.

For the application of degron systems in GFP-tagged lines, it is essential that the fluorophore does not interfere with the functionality of the fused protein and that both poly-peptides remain stably bound. We used CRISPR/Cas9 to trigger the homology directed repair (HDR) mechanism inserting the coding sequence of a flexible linker and *GFP* at the C-terminus of the endogenous *pmm2* locus, yielding a seamless Pmm2-GFP fusion (Fig. 1A). To increase single integration efficiency and enhance HDR, the PCR-donor sequence was protected with biotin at the 5′ end of each primer during amplification (Gutierrez-Triana et al., 2018). Following injections at the 1-cell stage, GFP fluorescence was used as a proxy for successful protein tagging. Offspring descending from positively screened embryos were validated by genotyping and raised. In the validated stable line we established, Pmm2-GFP expression was following the endogenous expression and was found ubiquitously and continuously (Fig. 1B; Fig. S1). Homozygous animals exhibited normal growth, development, and fertility, indicating that the GFP-tag did not interfere with Pmm2 function. A PMM2-enzyme assay performed on wild-type and Pmm2-GFP homozygous offspring revealed comparable enzyme activity, with a mean specific activity of 0.093 U/mg for wild-type and 0.095 U/mg for Pmm2-GFP (*n* total=93 of three independent replicates, respectively; Fig. 1C; Körner et al., 1998). Single copy integration of *GFP* into the *pmm2* locus was confirmed by PCR-genotyping and Southern blot analysis (Fig. S2). Sequencing of the *pmm2* cDNA reverse transcribed from mRNA isolated from the stable line confirmed the correct sequence and splicing of the transcript, resulting in a single open reading frame and seamless fusion of *pmm2* and *GFP* coding sequences in mature mRNA. This properly fused mRNA was transcribed and spliced from a modified genomic locus. The right homology flank (RH) of the donor was seamlessly integrated via HDR (Fig. 1A; Fig. S2). Interestingly correct splicing (and translation) occurred despite the partial duplication of the left homology flank (LH) [i.e. the entire coding sequence of exon 8 (ENSORLE00000306983) and parts of the leading intron]. Western blot analysis further confirmed the Pmm2-GFP fusion protein as the sole product from the tagged locus, without additional individual Pmm2 or GFP proteins (*n*=9 pooled hatchlings per genotype; Fig. 1D).

### The TIR1/mAID-GFP-nanobody degron system is effective but harbors high basal activity in medaka

**Fig. 2.**
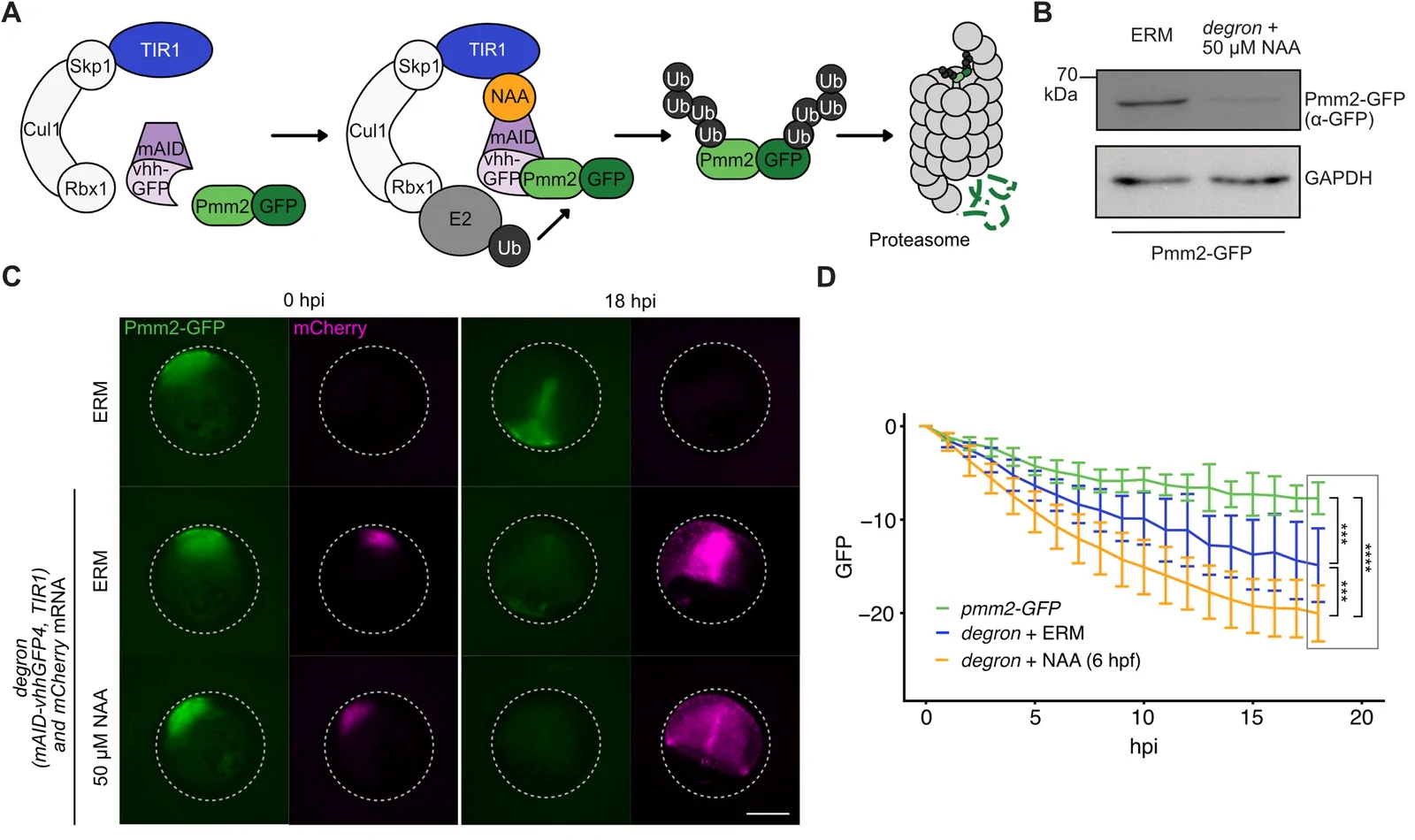
**TIR1 and mAID/GFP-nanobody degron system has strong basal activity in the absence of auxin in medaka.** (A) Schematic representation of an mAID and GFP-nanobody based, auxin inducible degron system (Daniel et al., 2018) planned for acute degradation of Pmm2-GFP in medaka. Upon auxin (NAA) induction, the E3 ubiquitin ligase complex SCF (Skp1, Cul1, F-box protein TIR1) dimerizes with the mAID-GFP nanobody, resulting in ubiquitination and degradation of GFP-tagged proteins by the proteasome. (B) Validation of Pmm2-GFP degradation via western blot analysis of stage 23 *pmm2-GFP* embryos comparing uninjected to degron-injected and induced (50 µM NAA) at 6 h post fertilization (hpf; *n*=35 pooled embryos per condition). Gapdh was used as loading control. (C) Time-lapse imaging of uninjected homozygous *pmm2-GFP* embryos and degron-injected embryos in ERM or induced with 50 µM NAA. (D) Quantification of mean GFP fluorescence following baseline correction over 18 h. Mean±s.d. are shown, one-way ANOVA test with Tukey’s post-hoc multiple comparison was performed on endpoints (grey box), adjusted *P*-values shown, ***P≤0.001, ****P≤0.0001. Uninjected homozygous *pmm2-GFP* (*n*=7), degron-injected in ERM (*n*=8) or induced with 50 µM NAA (*n*=25). Scale bar: 500 µm. hpi, hours post induction.

The combination of F-box protein OsTIR1 with nanobodies for degradation of GFP-fusion proteins in an auxin inducible manner was previously developed in human cell culture lines and applied in zebrafish embryos (Daniel et al., 2018). Here we attempted to target the endogenously tagged Pmm2-GFP with the vhhGFP4 nanobody to recruit it to Tir1 in an auxin inducible manner, thereby facilitating ubiquitination by the SCF complex (Fig. 2A). As the tolerated concentration of chemical effectors differs between organisms, an auxin (1-Naphthaleneacetic acid; NAA) toxicity test was first performed on wild-type medaka embryos. Embryos were subjected to a range of different NAA concentrations and phenotypes were assessed (Fig. S3). Embryos at high NAA concentrations (≥5 mM) displayed a wide range of developmental defects including blood clotting, edema around the heart or overall embryonic misdevelopment (Fig. S3). NAA incubations up to 0.5 mM did not lead to any scorable phenotypes.

To validate the functionality of the degron system in Pmm2-GFP medaka fish, the *TIR1* and *mAID-nanobody* mRNAs of the degron components were co-injected with *mCherry* mRNA as injection tracer into homozygous *pmm2-GFP* embryos at the 1-cell stage. A western blot analysis performed at stage 23 confirmed robust reduction of Pmm2-GFP protein (*n*=35 pooled embryos per condition; Fig. 2B). To analyze the kinetics of Pmm2-GFP degradation, the batch of injected embryos was separated and incubated with auxin or kept in embryo rearing medium (ERM) at 6 h post fertilization (hpf). GFP fluorescence was assessed continuously for 18 h (Fig. 2C). In uninjected *pmm2-GFP* controls (*n*=7), green fluorescence was continuously visible with a slight decrease over time (Fig. 2C,D, green curve). In the group induced with 50 µM NAA (*n*=25), the GFP expression quickly decreased (Fig. 2C,D, orange curve; Fig. S4). Degradation of GFP signals also occurred in the yolk of degron-injected and homozygous *pmm2-GFP* individuals, which highlights the acute degradation even of maternally provided Pmm2-GFP. Surprisingly, the GFP fluorescence in the degron-system injected but non-induced control group (*n*=8) dropped as well (Fig. 2C,D, blue curve; Fig. S4), indicating leakiness of the employed TIR1/mAID system in medaka, i.e. degradation in the absence of auxin.

Although reduction increased by incubation with NAA (Fig. 2C,D; Fig. S4), this confirmed that the degron system, even without NAA induction, harbors a strong basal degradation activity. Due to the leakiness, the TIR1/mAID system was not suitable for use as an acute knockdown application.

### Efficient inducible Pmm2-GFP depletion by the improved TIR1(F74G)/mAID-GFP-nanobody degron system

**Fig. 3.**
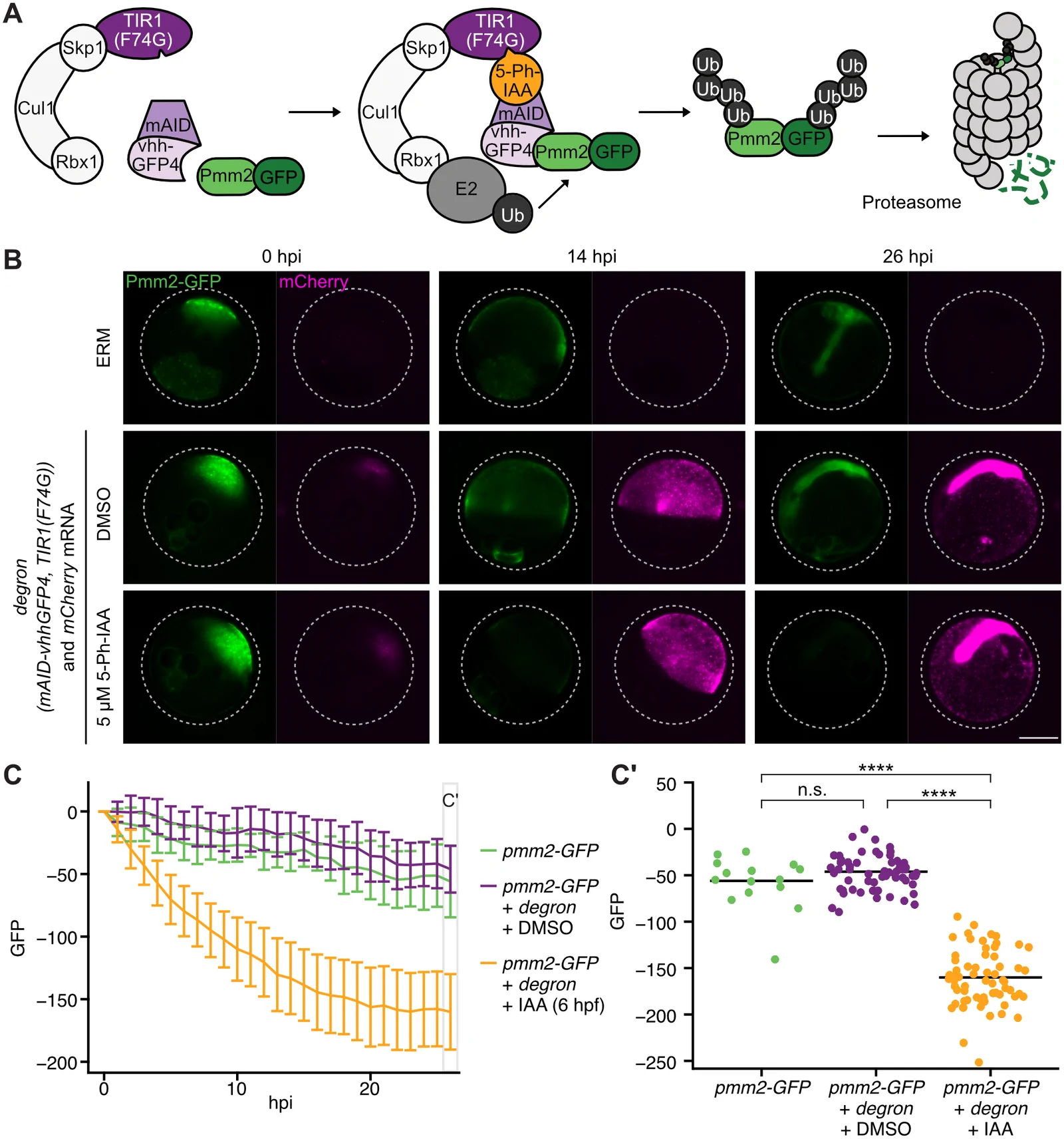
**Combination of TIR1(F74G) with mAID/GFP nanobody as degron system enables acute and inducible knockdown of Pmm2-GFP in medaka.** (A) Schematic representation of the TIR1(F74G) variant combined with the mAID/GFP-nanobody (vhhGFP4) based degron (Daniel et al., 2018; Yesbolatova et al., 2020) to induce degradation of Pmm2-GFP in an auxin-analog (5-Ph-IAA) inducible manner in medaka. (B) Time-lapse imaging of uninjected *pmm2-GFP*, degron-injected *pmm2-GFP* embryos incubated in ERM or 5 µM 5-Ph-IAA at 6 h post fertilization (hpf). (C) Quantification of mean GFP fluorescence following baseline correction over 26 hpi. Mean±s.d. of triplicates shown, uninjected *pmm2-gfp* control (*n* total=15; green), *degron/mCherry* injected, non-induced embryos (*n* total=61; purple), induced (*n* total=65; orange). (C′) Scatterplot of raw data at 26 hpi. Mean is shown as black line, one-way ANOVA test with Tukey’s post-hoc multiple comparison was performed, adjusted *P*-values shown, ns, *P*>0.05, ****P≤0.0001. Scale bar: 500 µm. hpi, hours post induction; 5-Ph-IAA, auxin analog.

To overcome leakiness of the TIR1 and mAID/GFP-nanobody degron system, we exchanged the TIR1 F-Box protein with a recently reported variant (F74G), mitigating basal degradation of mAID-tagged proteins (Fig. 3A) (Yesbolatova et al., 2020). The F74G mutation enables the TIR1 protein to bind the auxin analog 5-phenyl-indole-3-acetic acid (5-Ph-IAA) instead of the canonical NAA. Since 5-Ph-IAA can be used at very low concentrations, it minimizes potential toxic effects of this compound. A toxicity screen with varying concentrations of 5-Ph-IAA indeed demonstrated normal embryonic development in medaka fish embryos (Fig. S3).

To test the specificity and efficiency of TIR1(F74G) to bind the mAID-GFP-nanobody in the presence of 5-Ph-IAA, degron components [*TIR1(F74G)* and *mAID-GFP-nanobody*] were injected as mRNA into 1-cell stage embryos of the homozygous *pmm2-GFP* line along with the injection tracer *mCherry*. The injected embryos were split into treated (5 µM 5-Ph-IAA; *n* total=65) and untreated (*n* total=61) groups (Table S1).

Green (Pmm2-GFP) fluorescence was acquired by time-lapse imaging over 26 hpi (Fig. 3B). In the uninjected *pmm2-GFP* control groups (*n* total=15), green fluorescence was continuously visible with a slight decrease over time (Fig. 3B,C, green curve; Fig. S5). In the degron-injected but non-induced groups, the GFP fluorescence followed the same trend as the uninjected control, indicating no obvious basal degradation of the TIR1(F74G)/mAID-vhhGFP4 system (Fig. 3B,C, purple curve; Fig. S5). An immediate decrease in GFP fluorescence was observed in the group induced with 5 µM 5-Ph-IAA, reaching a plateau about 20 h post induction (Fig. 3B,C, Fig. S5). Endpoint analysis of GFP levels showed high homogeneity of degradation efficiency (Fig. 3C′). The robustness of the mRNA injection protocol ensures efficient degradation with low levels of inter-individual variability. This is apparent in the non-correlation of mCherry and GFP expression levels at the endpoint of analysis (Fig. S6). Based on fitted curve analysis, Pmm2-GFP was degraded with a half-life of approximately 6.9 h (Fig. S7).

**Fig. 4.**
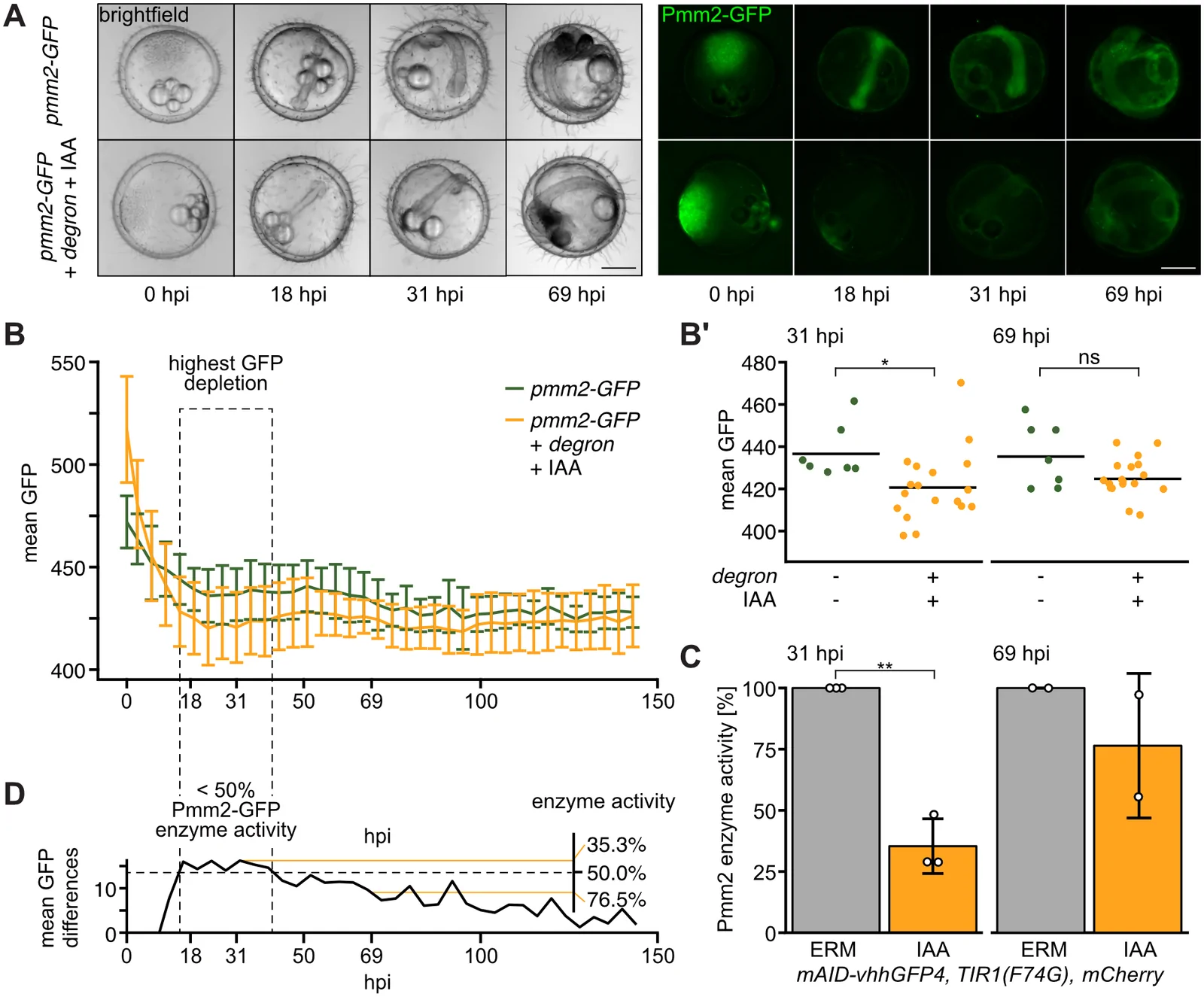
**Recovery of Pmm2-GFP expression and enzyme activity.** (A) Time-lapse imaging of uninjected *pmm2-GFP* and degron-injected *pmm2-GFP* embryos incubated in 5 µM 5-Ph-IAA (lower row) from 6 h post fertilization on. (B) Quantification of mean GFP fluorescence over 143 hpi. Mean±s.d. shown, uninjected *pmm2-gfp* control (*n*=7), *degron/mCherry* injected, induced (*n*=18). (B′) Scatterplot of individual mean GFP levels at 31 hpi and 69 hpi. Mean is shown as black line, *t*-test, ns, *P*>0.05, *P≤0.05. (C) Pmm2 enzyme activity assay comparing control (injected, non-induced; grey) and degron-injected and induced (5 µM 5-Ph-AA) embryos (orange). Three independent experiments with lysates of *n*=25, 26 and 34 pooled embryos for 31 hpi. Two independent experiments with lysates of pooled embryos in ERM (*n*=19 and 23) and in 5-Ph-IAA (*n*=12 and 32) for 69 hpi. Mean±s.d. are shown, *t*-test, **P≤0.01. (D) Differential GFP levels of uninjected and injected and depletion induced specimens from B plotted and correlated with measured Pmm2-GFP enzyme activity at 31 and 69 hpi from C. Estimated window of Pmm2-GFP activity below 50% indicated (dashed grey line). Scale bars: 500 µm. hpi, hours post induction; 5-Ph-IAA, auxin analog.

To assess the dynamics of the Pmm2-GFP degradation and recovery, we performed extended time-lapse imaging of GFP fluorescence over 143 h post induction with 5 µM 5-Ph-IAA at 6 hpf and biochemical Pmm2 enzyme activity assays (Fig. 4).

Gross morphological and GFP fluorescence analysis revealed dynamic loss and recovery of GFP fluorescence without apparent morphological phenotypes in degron-injected and induced embryos (Fig. 4A; Fig. S8, *n*=7 non-injected *pmm2-GFP* embryos and *n*=18 degron-injected and 5-Ph-IAA induced embryos). Detailed analysis of the Pmm2-GFP dynamics indicated a temporal window of highest depletion of Pmm2-GFP (Fig. 4B). Comparing Pmm2-GFP control embryos (green line) to degron-injected and induced (5 µM 5-Ph-IAA) embryos (orange line) highlighted a significant difference in GFP levels at 31 hpi, while after recovery (at 69 hpi), GFP levels did not differ significantly (Fig. 4B′).

When induced at later stages (24 hpf), Pmm2-GFP degradation was still observed however less pronounced compared to induction at earlier time points (Fig. S9, *n*=12 uninjected *pmm2-GFP* embryos*, n*=24 degron-injected and non-induced *pmm2-GFP* control embryos and *n*=25 degron-injected and induced embryos).

To correlate GFP fluorescence levels to Pmm2-GFP enzyme activity, we measured Pmm2 enzyme activity at 31 hpi and 69 hpi in degron-injected and non-induced versus degron-injected and induced embryos. Within the window of maximum GFP depletion (31 hpi), net Pmm2 activity was reduced to 35.3% (three independent replicates with lysates of *n*=25, 26 and 34 pooled embryos) and Pmm2 activity recovered to 76.5% at 69 hpi [two independent replicates with pooled embryo lysates (control: *n*=19 and 23; 5-Ph-IAA: *n*=12 and 32)] (Fig. 4C). Consistent with observed GFP fluorescence recovery, the net Pmm2 enzyme activity at 69 hpi was clearly regained although statistical testing could not be performed (based on the two independent replicates).

Recovery of the Pmm2-GFP level became apparent as the GFP intensity between the depleted and control groups converged over time, i.e. the difference between mean GFP intensities reduced (Fig. 4D). Correlating these differential GFP fluorescence levels to the measured Pmm2 enzyme activity at 31 and 69 hpi, we estimated a temporal window of Pmm2 activity below 50% between 15 and 41 hpi (Fig. 4D). This narrow window of protein knockdown appears to be too short to cause sustained phenotypic effects but is especially relevant for capturing acute molecular changes. For example, proteomic analysis during this interval could reveal proteins affected by reduced Pmm2 enzyme activity.

The combined use of TIR1(F74G) and mAID-GFP-nanobody thus allowed acute and dynamic knockdown of an endogenously GFP-tagged protein in a developing medaka embryo. In the case of Pmm2-GFP, this system reduced the net enzyme activity of Pmm2 to likely pathogenic levels reported from PMM2-CDG patients during early development (Barone et al., 2015; Grünewald et al., 2001; van Schaftingen and Jaeken, 1995; Vals et al., 2017).

Taken together, we show that an mRNA-based systemic degron-induced knockdown setup offers temporal control of protein abundance immediately upon induction, reducing zygotic as well as maternal Pmm2-GFP in medaka. The kinetics of the system revealed fast depletion of Pmm2-GFP with a strong ubiquitous protein degradation and 6.9 h half-life after induction at non-toxic concentration of 5-Ph-IAA. Especially in the context of disease modeling that aims for early developmental stages or rapid protein turnover, the system is well suited to model the reduction, severe impairment or loss of protein function associated with disease progression.

For Pmm2-GFP, this opens the possibility to generate a hypo-glycosylation environment at early stages of development, which is challenging to accomplish with classic genetic methods that rely on *pmm2* mutant alleles. Homozygous animals often cannot be raised to maturity due to their severe pathology. Heterozygous breeds in contrast are usually not haploinsufficient and enzyme levels exceed the phenotypic threshold. Homozygous offspring of heterozygous mothers in turn do not allow us to study the early onset of CDG-phenotypes, as Pmm2 is maternally contributed, mitigating the early embryonic phenotype.

Comparing previous reports of different TIR1/AID systems and its genetic variants highlight that there is not a single solution generally applicable across different model systems and that TIR1/AID is frequently reported to show basal activity (Schiksnis et al., 2020). The system was improved by mutating the phenylalanine in position 74 in the TIR1 protein, resulting in different efficiency depending on the organism used (*Drosophila melanogaster*, *Caenorhabditis elegans*, *Oryzias latipes*, *Schizosaccharomyces pombe*) (Goldner et al., 2023; Hills-Muckey et al., 2022; Kiyomitsu et al., 2024; Zhang et al., 2022). In yeast, a phenylalanine to alanine change is reported to be more effective compared to other mutations (Ogawa et al., 2023). In cell culture systems, comparable levels of protein degradation were observed for both F74A and G variants. However, F74A showed higher activity with IAA, preferring the usage of F74G variant in mouse (Yesbolatova et al., 2020). Further, there is a clear improvement when using the TIR1-variant sensitive to auxin analogs (5-Ad-IAA; 5-Ph-IAA) versus the native plant hormone when it comes to toxicity effects of the amount required for the induction of protein knockdown (Nishimura et al., 2020; Yesbolatova et al., 2020). In the end it is advisable to test reported alternatives like the TIR1(F74G)/mAID-GFP-nanobody system, as it may vary in basal activity or effectivity in the used species or cell system.

While the acute Pmm2 protein degradation clearly impacted on enzymatic activity, it did not result in a scorable morphological phenotype. Our system relies on an mRNA injection approach, therefore, the availability and activity of degron components declines over time as mRNA and proteins face natural turnover. At the same time the developing embryo is constantly supplying new Pmm2-GFP protein. Thus, these dynamics create a ‘natural’ reversibility, leveling out the degradation. This is in contrast to systemic approaches that cause clear morphological phenotypes in vertebrate animal models (Chan et al., 2016; Cline et al., 2012; Himmelreich et al., 2015; Klaver et al., 2021; Schneider et al., 2012; Thiel et al., 2006), highlighting the potential for mapping critical periods with acute knockdown approaches.

For developmentally relevant time windows requiring acute protein degradation, the mRNA-based approach provides many advantages. It gives immediate access to any endogenously GFP tagged target protein and at the same time it overcomes the need for universal, ubiquitous promoters active during early embryogenesis. The degradation efficiency can immediately be adjusted (by the amount of injected mRNA) to the levels of expression of the targeted protein of interest, a requirement that is hard to meet in fixed and stable transgenic settings. The combination of tunable (LexPR) and ubiquitous promotors driving the degron components may provide a solution to address later developmental and subsequent stages.

The system presented thus offers a fast and immediate approach for highly efficient acute protein degradation of developmentally relevant proteins and provides an important complementation to genetic targeted inactivation, in particular in settings with a strong maternal contribution.

## MATERIALS AND METHODS

### Fish maintenance

Medaka (*O.* *latipes*) stocks were maintained (fish husbandry, permit number 35–9185.64/BH Wittbrodt) and experiments (permit number 35–9185.81/G-271/20 Wittbrodt) were performed in accordance with local animal welfare standards (Tierschutzgesetz §11, Abs. 1, Nr. 1) and European Union animal welfare guidelines (Bert et al., 2016). Fish were maintained in closed stocks and constant recirculating systems at 28°C on a 14 h light/10 h dark cycle. The fish facility is under the supervision of the local representative of the animal welfare agency.

### Line generation

The endogenously tagged *pmm2-GFP* line was generated as described in Gutierrez-Triana et al. (2018). In short: the *pmm2* template plasmid containing GFP sequence flanking 5′ and 3′ homology flanks (HFs) was created via GoldenGATE cloning described in Kirchmaier et al. (2015). Biotinylated primers were used to amplify a modified PCR donor. To minimize interference of Pmm2 stability and folding capacity, a flexible GS linker composed of 21 amino acids upstream of GFP (Sabourin et al., 2007) was used. Wild-type Cab embryos were injected with an sgRNA targeting the *pmm2* locus at the C-terminus (5′-TCTTCTTCTGCTGAAGCTAC[TGG]-3′ and 5′-TCTTCTGCTGAAGCTACTGG[AGG]-3′), *heiCas9* mRNA, and *mCherry* mRNA as injection tracer. Positively screened embryos were raised to adulthood and outcrossed to wild-type medakas. The resulting generation was screened for ubiquitous GFP expression. Founder fish were outcrossed to raise stable heterozygous individuals, following generation of a fertile homozygous *pmm2-GFP* line. Single integration was verified by Southern blot, PCR genotyping, and western blot (Fig. S1).

### Genotyping – gDNA and cDNA analysis

For gDNA extraction from embryos, up to five individuals were ground with plastic pestles in 100 μl Fin-Clip buffer [0.4 M Tris-HCl pH 8.0, 5 mM EDTA pH 8.0, 0.15 M NaCl, 0.1% SDS in distilled water] and 5 µl Proteinase K (20 mg/ml), incubated for 1 h at 60°C. After addition of 200 µl nuclease free water, lysates were incubated at 95°C for 20 min. Samples were stored at 4°C. Genotyping was performed using 1 μl of lysate in a 50 μl PCR reaction with 1x Q5 reaction buffer (New England Biolabs, NEB), 200 µM dNTPs (Sigma-Aldrich), 200 µM per primer (Eurofins Genomics) and 0.012 U/µl Q5 polymerase (NEB). *Pmm2* locus primers were used: *Pmm2-fwd* 5′-TGGACGACACAGATGATGCT and *Pmm2-rev* 5′-CTCACAGACTGACCCTCACC. PCRs were run for 30 cycles (30 s, 98°C denaturation; 30 s, 67°C annealing, 90 s, 72°C extension). Amplicons were size fractioned by gel electrophoresis on 1% agarose gel in 1x TAE (40 mM Tris, 20 mM acetic acid, 1 mM EDTA) and sent for Sanger sequencing (Eurofins Genomics) after gel extraction and purification (monarch kit, NEB).

For RT-PCR, total RNA was extracted from five *pmm2-GFP* and wild-type hatchlings by lysis in TRIzol (Ambion) and purification with the Direct-zol RNA MicroPrep Kit (Zymo Research) according to the manufacturers protocol. The complementary DNA (cDNA) was prepared by reverse transcription using Revert Aid Kit (Thermo Fisher Scientific). Primers binding to exon 2 of *Pmm2* (5′- AAACGCAGAGGCTCAGGACTCG) and the 3′UTR (5′- TGAGGTCACATCCCGTGTTG) were used for a 30 cycles PCR as described above (68°C annealing, 2 min 72°C extension). PCR products were run on a 1.5% agarose gel in 1x TAE and sent for Sanger sequencing after filter-column purification from the gel (Monarch, NEB).

### Protein extraction, quantification and immunoblot analysis for Pmm2-GFP line validation

For validation of the Pmm2-GFP line, protein lysates (*n*=9 pooled hatchlings per lysate for each genotype) for immunoblot analysis were prepared from hatchlings homogenized in 1x PBS (137 mM NaCl, 2.7 mM KCl, 1.44 g/l Na2HPO4, 240 mg/l KH2PO4) with 1% TritonX with 1x cOmplete EDTA–free Protease Inhibitor Cocktail (Roche). After incubation for 20 min on the rotator in the cold room, samples were centrifuged at 12,000 ***g*** at 4°C for 10 min. The supernatant was transferred to a low protein binding Eppendorf tube. The concentrations of the lysates were determined with BCA protein-assay kit (23225, Thermo Fisher Scientific). 25 µg of protein lysate was boiled in 2.5x Laemmli sample buffer [157.5 mM Tris-HCl (pH 6.8), 5% SDS, 25% glycerol, 12.5% 2-mercaptoethanol, 0.08% Bromophenol Blue] for 10 min at 95°C and stored at −80°C. The samples were run on a self-made SDS-PAGE with 10% separation gel and 4% stacking gel at 60 V for the first 30 min and at 120 V afterwards. Transfer of the proteins to a PVDF membrane (IPVH00010, Millipore Immobilon-P) was done at 350 mA for 1 h at 4°C. Membranes were blocked in 5% Skim Milk (Sigma-Aldrich) in 1xTBST [50 mM Tris-HCl (pH 7.5), 150 mM NaCl, 0.1% (v/v) Tween 20] for 1–2 h at room temperature (RT). Blots were incubated with primary antibody in blocking solution over night at 4°C. Antibodies used: anti-GFP (A11122, Invitrogen, 1:500), anti-PMM2 (Proteintech, 10666-1-AP, 1:500), anti-GAPDH (Cell Signaling Technology, 14C10C21188, 1:1000). Blots were washed 4x 10 min with 1x TBST and incubated afterwards with corresponding secondary antibody [goat anti-rabbit, Santa Cruz Biotechnology (sc-2005), 1:10000] diluted in 1x TBST for 1 h at RT. Blots were washed with 1x TBST 4x 10 min. Proteins were visualized with ECL Substrate (Pierce ECL Western Blotting Substrate) on a Gel Doc Imager (INTAS, Göttingen).

### Southern blot

To validate single integration of *GFP* into the *pmm2* locus, Southern blot was performed on gDNA extracted from a homozygous *pmm2-GFP* adult head. gDNA was isolated by ethanol precipitation after phenol-chloroform extraction and resuspended in 1x TE buffer (10 mM Tris HCl pH 8.0, 1 mM EDTA pH 8.0). 10 µg *pmm2-GFP* gDNA and 200 pg of *GFP* control plasmid were digested with 10 U of BspHI (NEB) in combination with 20 U of HindIII (NEB) or 20 U of BsaI HF (NEB) at 37°C overnight. The digested fragments were size fractionated by electrophoresis on a 0.8% agarose gel in 1x TAE buffer. On the gel, DNA was depurinated in 0.25 N HCl for 30 min at RT, rinsed with H_2_O, denatured in 0.5 N NaOH, 1.5 M NaCl solution for 30 min at RT and neutralized in 0.5 M Tris HCl, 1.5 M NaCl, pH 7.2 before being transferred overnight at RT onto a Hybond-N+ membrane (Sigma-Aldrich) by capillary transfer. The membrane was washed with 50 mM NaPi for 5 min at RT, DNA was UV-crosslinked and pre-hybridized in Church hybridization buffer (0.5 M NaPi, 7% SDS, 1 mM EDTA pH 8.0) at 65°C for 30 min. Probe synthesis was performed with PCR DIG Probe Synthesis Kit (Roche) according to the manufacturer's protocols (primers used: GFPprobe-fwd 5′-GTGAGCAAGGGCGAGGAGCT, GFPprobe-rev 5′-TTACTTGTACAGCTCGTCCATG) and PCR conditions: initial denaturation at 95°C for 2 min, 35 cycles of 95°C for 30 s, 60°C for 30 s, 72°C for 40 s and final extension at 72°C for 7 min. The probe comprised a 700 bp fragment containing *GFP* sequence. The probe was denatured in hybridization buffer for 10 min at 95°C and hybridized with the membrane overnight at 65°C. The membrane was washed with pre-heated (65°C) Church washing buffer (40 mM NaPi, 1% SDS) at 65°C for 10 min, continued at RT for 10 min and washed with 1× DIG1 and 0.3% Tween (Sigma-Aldrich) for 5 min at RT. The membrane was blocked in 1% w/v blocking reagent (Roche) in 1x DIG1 solution at RT for at least 30 min. The membrane was incubated with 1:10,000 anti-digoxigenin-AP Fab fragments (Roche) for 30 min at RT in 1% w/v blocking reagent (Roche) in 1x DIG1 solution. Two washing steps with 1x DIG1 and 0.3% Tween were performed for 20 min at RT followed by a 5 min washing step in 1x DIG3 (0.1 M Tris pH 9.5, 0.1 M NaCl) at RT. Detection was performed using 6 µl/ml CDP star (Roche) and chemiluminescence was acquired on an Intas Imager (INTAS, Göttingen).

### Auxin toxicity test

For the auxin toxicity test a 0.1 M stock solution was prepared by dissolving NAA powder (NAA, sodium 1-naphtaleneacetate, Santa Cruz Biotechnology) in embryo-rearing medium (1× ERM: 17 mM NaCl, 0.4 mM KCl, 0.27 mM CaCl2•2H2O, 0.66 mM MgSO4•7H2O and 17 mM HEPES). Working concentrations were further diluted with ERM from the stock solution. Wild-type Cab embryos from adult crosses were collected and kept until stage 10 (∼6 hpf) in ERM. Next, embryos were induced with 0.1 mM, 0.5 mM, 1 mM, 5 mM and 10 mM NAA or kept in ERM (*n*=25 for each concentration) and incubated at 26°C. Auxin solution was refreshed every 24 h. The resulting phenotypes were assessed by imaging every day until 4 dpf under a stereomicroscope with an attached Nikon DXM1200 digital camera.

For the auxin analog 5-Ph-IAA toxicity test, the powder (MedChemExpress, #HY-134653) was dissolved in DMSO and a 10 mM stock solution was prepared in 1x PBS. Working concentrations were further diluted with ERM from the stock solution. Wild-type Cab embryos from adult crosses were collected and kept until stage 10 (∼6 hpf) in ERM. Next, embryos were induced with 1 µM (*n*=19), 2.5 µM (*n*=19), 5 µM (*n*=20), 10 µM (*n*=19) 5-Ph-IAA and as control in 0.005% DMSO (*n*=20) and ERM (*n*=21) and incubated at 26°C. Auxin solution was refreshed every 48 h. The resulting phenotypes were assessed by imaging every day until hatch under a stereomicroscope with the attached Nikon camera.

### Degron system plasmids and mRNAs synthesis

The degron system mRNAs were synthesized from constructs (Daniel et al., 2018) cloned into pCS2+ plasmids. pCDNA5FRT/TO_HA-mAID-nanobody was a gift from Joerg Mansfeld (Addgene plasmid # 117713; http://n2t.net/addgene:117713; RRID:Addgene_117713) and pCS2+_Flag-myc-NES-Tir1 was also a gift from Joerg Mansfeld (Addgene plasmid # 117717; http://n2t.net/addgene:117717; RRID:Addgene_117717). TIR1F74G point mutation (Yesbolatova et al., 2020) was introduced via Q5 mutagenesis.

For *in vitro* transcription, 5 µg of the plasmids were linearized O/N at 37°C and the digests were purified with QIAquick PCR Purification Kit (Qiagen). *In vitro* transcriptions of mRNAs were prepared with the mMESSAGE mMACHINE SP6 Transcription Kit (Invitrogen) and purified with the RNeasy Mini Kit (Qiagen), according to manufacturers' protocols. The mRNA quality was assessed through an RNA agarose test gel.

### Microinjections

For the validation of degron systems, microinjections were performed in homozygous *pmm2-GFP* embryos at the one-cell stage. Freshly fertilized eggs were injected with respective injection mixes. Injection mixes and numbers of total injected embryos, excluded embryos, dead embryos and analyzed embryos are provided in Table S1. The injection amount and concentration of the degron components were chosen at saturating levels.

After injections, embryos were transferred into fresh 1× ERM and incubated at 26°C. 6 or 24 h after fertilization embryos were induced with auxin or the auxin analog 5-Ph-IAA. Prior to induction, dead and mCherry-negative embryos were discarded.

### Time-lapse acquisition and quantification of Pmm2-GFP degradation

Time-lapse images of degron-injected *pmm2-GFP* embryos were acquired on a ACQUIFER imaging Machine (Bruker, Heidelberg, Germany). Embryos were placed in a 96-well plate with 200 µl media and inserted into the plate holder of the imaging machine. Imaging was performed at 26°C for 18, 26 or 143 h. A set of five z-slices (300 µm each slice) was acquired in bright field, 561 nm and 470 nm fluorescence channels with 2x NA 0.06 objective (Nikon, Düsseldorf, Germany) every 2 or 4 h.

For analysis of the degron kinetics a self-written Fiji (Schindelin et al., 2012) macro was used. In brief, for each timepoint, a maximum projection of the z-stack was calculated for both fluorescence images. Next, the maximum-projection images were cropped by a rectangular mask in which the embryos lie in the middle to exclude background. The fluorescence intensity from each embryo for each time-point was used for analysis of degradation kinetics of GFP. The mean GFP was baseline normalized, i.e. subtracting the initial time zero value from every point in the curve.

The plots were visualized within RStudio (version 2024.4.2.764; Posit team, 2024). Statistical analysis was also performed in RStudio.

### Validation of degradation via western blot

Pmm2-GFP protein degradation was validated via western blot. For sample preparation, stage 23 embryos (*n*=35 pooled embryos per condition) were gently rolled on sandpaper to remove the outer hair and treated afterwards in glass vials with hatching enzyme for ∼45 min at 28°C. Next, embryos were washed 4x with ERM and transferred into a glass dish filled with ERM. Remnants of the chorion were removed, and the yolk was torn open with forceps. Samples were centrifuged for 2 min at 956 ***g*** at 4°C. Afterwards, the supernatant was removed, and the samples were smashed in lysis buffer with a plastic pestle. The subsequent procedure for protein lysates and western blot analysis, was as described previously for the preparation of protein lysates from hatchlings in the ‘Protein extraction, quantification and immunoblot analysis for *pmm2-GFP* line validation’ section.

### Pmm2 enzyme activity assay

Protein lysates were extracted from stage 23 (31 h post induction) or stage 29 (69 h post induction) degron-injected *pmm2-GFP* embryos and the procedure was the same as described above in the ‘Validation of degradation via western blot’ section. Three independent measurements with lysates from pooled embryos (wild-type Cab and *pmm2-GFP: n*=25, 34, and 34 and degron-injected *pmm2-GFP* in ERM or 5-Ph-IAA: *n*=25, 26, and 34) were taken at stage 23. Two independent measurements with lysates from pooled embryos in ERM (*n*=19 and 23) and in 5-Ph-IAA (*n*=12 and 32) were taken at stage 29. After the last centrifugation step, the samples were snap-frozen in liquid nitrogen and stored at −80°C. The sample pellets were resuspended in lysis buffer, which contained protease inhibitors without detergent. Measurement of the Pmm2 activity followed the procedure of Körner et al. (1998). In short, 30 μg of protein were mixed with NADP, MgCl_2_, glucose-1-phosphate, mannose-6 phosphate isomerase (MPI) and phosphoglucose isomerase (PGI). In order to use Pmm2 as a rate-limiting step of the reaction, both enzymes, MPI and PGI, were supplemented in excessive amount to oversaturate them. The conversion of NADP to NADPH was measured at 340 nm for 2 h. Samples were measured in triplicates. The bar plots were visualized and statistical analysis was performed within RStudio (version 2024.4.2.764; Posit team, 2024).

## Supplementary Material

10.1242/biolopen.062081_sup1 Supplementary information

## Data and resource availability

All relevant data and details of resources can be found within the article and its supplementary information.
