# Supplementary material for: Establishing an auxin-inducible GFP nanobody-based acute protein knockdown system to mimic hypomorphic mutations during early medaka embryogenesis
Source: Biol Open. 2025 Nov 7;14(11):bio062081. doi: 10.1242/bio.062081 (PMC12641487; doi:10.1242/bio.062081)
Supplement: Supplementary information [file biolopen-14-062081-s1.pdf]

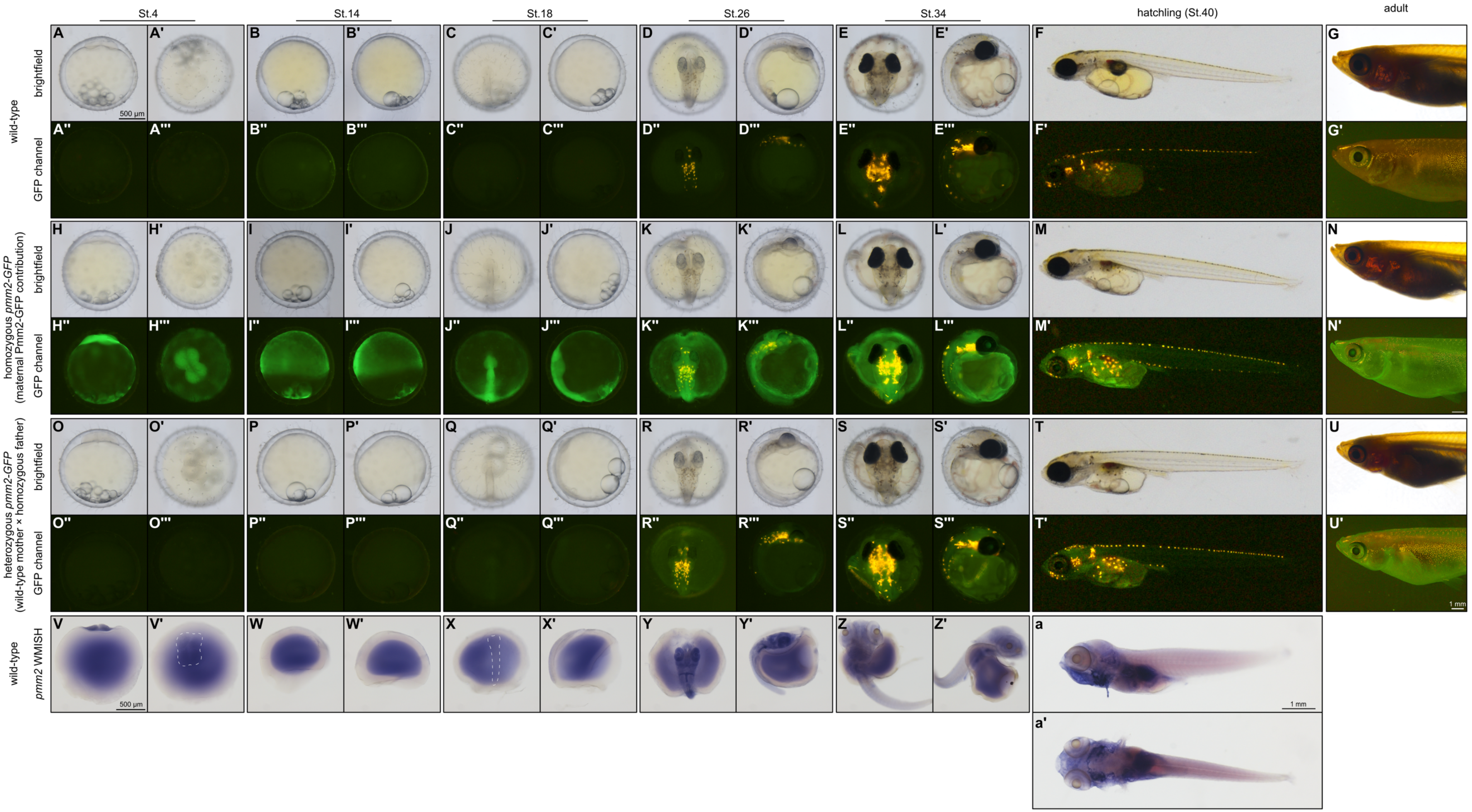

Fig. S1. Expression analysis of *pmm2-GFP* tagged line across development. A-U) Brightfield and GFP channel pictures across embryonic developmental stages, hatchling and adult in wild type (A-G), homozygous offspring with maternal *pmm2-GFP* contribution (H-N) and heterozygous offspring derived from outcross with wild-type mother (O-U). Developmental stages compared to whole mount in situ (WMISH) analysis of wild-type *pmm2* expression. Note high mRNA content in 4-cell stage due to maternal contribution. Zygotic pmm2-GFP expression increases during neurulation (cf. stage 26 R and Y). Yellow, red autofluorescence of pigments visible in green channel (≥ St. 26). Scale bar 500 μM or 1 mm. St., stage

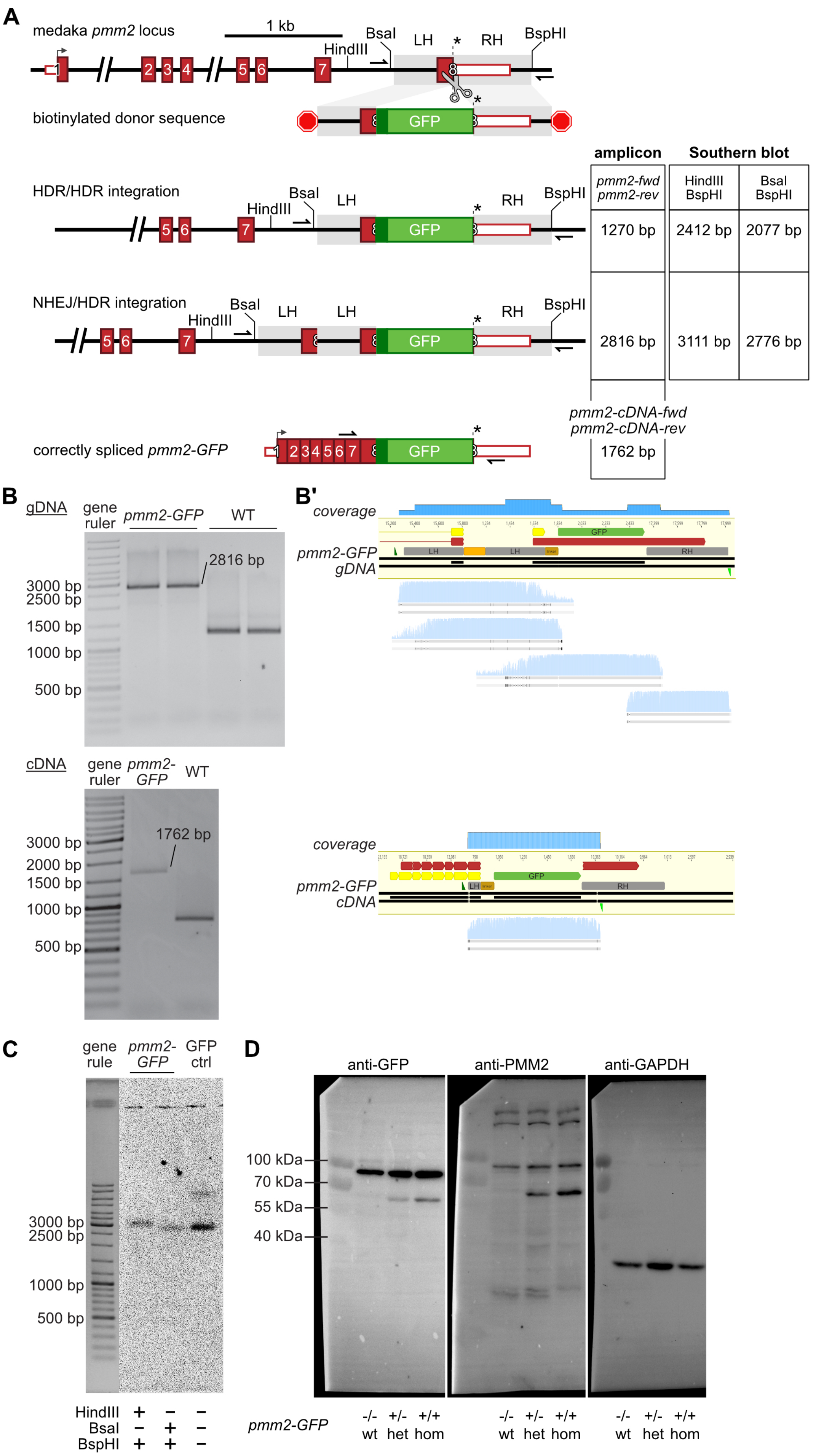

Biology Open • Supplementary information

Fig. S2. Validation of *pmm2-GFP* tagged line. A) Possible outcomes of GFP integration into Pmm2 locus, their sizes after locus-locus PCR and restriction enzyme cutting sites for southern blot analysis. B) gDNA and cDNA sequencing of homozygous *pmm2-GFP* hatchlings. Locus-locus PCR on gDNA indicates NHEJ event. Sanger sequencing confirms 5' NEHJ and 3'HDR integration (red – mRNA, yellow - CDS). C) Southern blot analysis of homozygous *pmm2-GFP* adults confirm single GFP integration. Single band hybridization signals were detected when cutting outside and within the 3'HF with two sets of enzymes (BsaI/BspHI and HindIII/BspI). D) Whole western blots from analysis of lysates from *pmm2*/*pmm2* wild-type, *pmm2*/*pmm2-GFP* heterozygous and *pmm2-GPF/pmm2-GFP* homozygous hatchlings from Fig. 1. Pmm2 and GFP specific antibodies were used and Gapdh was used as loading control. cDNA, complementary DNA; gDNA, genomic DNA; HDR, homology directed repair; het, heterozygous; hom, homozygous; NHEJ, non-homologous end-joining; wt, wild-type

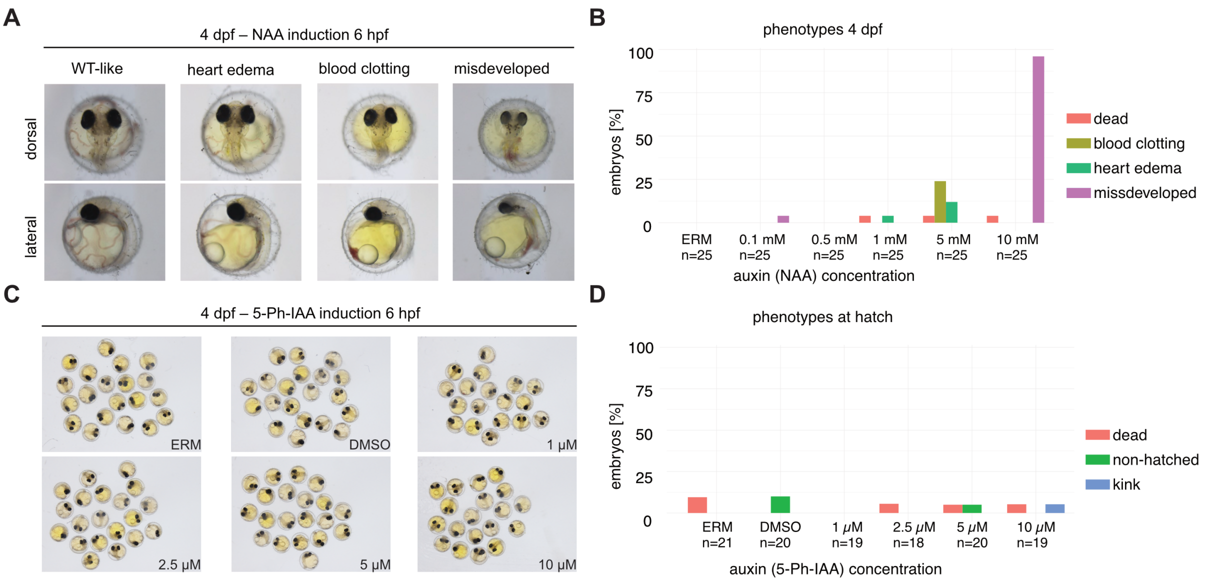

Fig. S3. Auxin toxicity in wild-type embryos. A) Representative NAA induced phenotypes resulting from incubation in auxin solutions 6 hpf with different concentrations. Embryos were incubated in 0.1 mM, 0.5 mM, 1 mM or 10 mM NAA and controls in ERM (n = 24 for each concentration). B) Quantification of NAA induced phenotypes from an auxin (NAA) toxicity test in wild-type embryos 4 dpf. C) 5-Ph-IAA induced phenotypes resulting from incubation in auxin solutions with different concentrations 6 hpf. Embryos were incubated in 1 μM (n = 19), 2.5 μM (n = 19), 5 μM (n = 20) or 10 μM (n = 19) 5-Ph-IAA and controls incubated in 0.005% DMSO (n = 20) or kept in ERM (n = 21). D) Quantification of 5-Ph-IAA induced phenotypes from toxicity test at hatch. dpf, days post fertilization; hpf, hours post fertilization; NAA, auxin; WT, wild-type

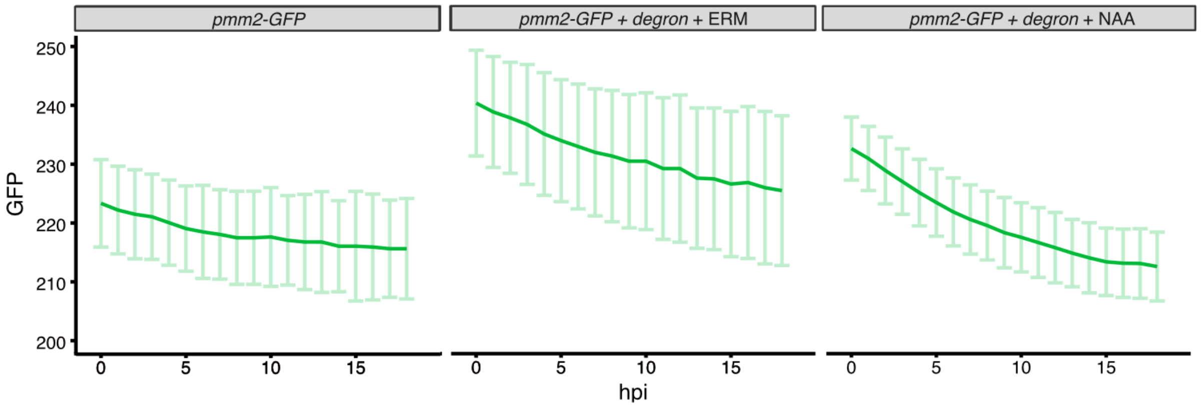

Fig. S4. Mean GFP fluorescence acquisition of control and degron-injected group. Temporal fluorescence acquisition in uninjected homozygous *pmm2-GFP* embryos (n=7), degron-injected control in ERM (n = 8) and degron-injected and induced with 50 μM NAA group (n = 25). Mean values ± sd. Rawdata to data shown in Fig. 2D. hpi, hour post induction; NAA, auxin

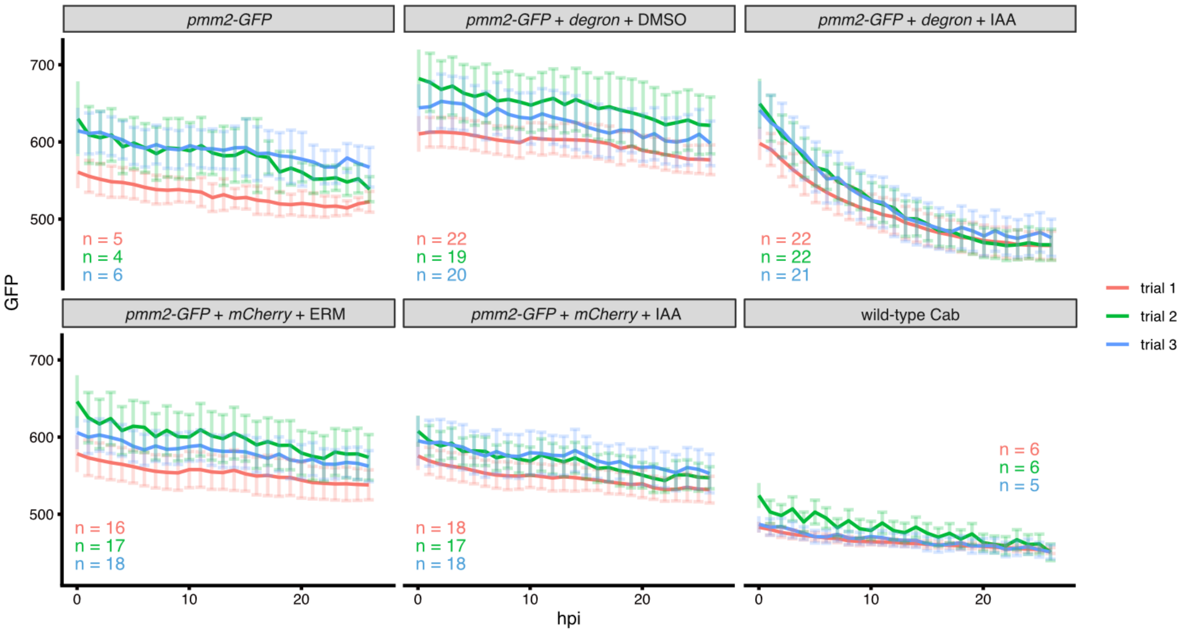

Fig. S5. Quantification of mean GFP fluorescence of control and degron-injected groups. Temporal mean GFP fluorescence acquisition over 26 hours in control, degron-injected and treated groups. Mean values ± sd given per trial. Uninjected *pmm2-GFP* embryos (trial 1: n = 5, trial 2: n = 4, trial 3: n = 6), degron-injected *pmm2-GFP* embryos in 0.005 % DMSO (trial 1: n = 22, trial 2: n = 19, trial 3: n = 20), degron-injected *pmm2-GFP* embryos induced with 5 μM IAA (trial 1: n = 22, trial 2: n = 22, trial 3: n = 21), mCherry-injected *pmm2-GFP* embryos in ERM (trial 1: n = 16, trial 2: n = 17, trial 3: n = 18), mCherry-injected *pmm2-GFP* embryos in 5 μM IAA (trial 1: n = 18, trial 2: n = 17, trial 3: n = 18) and wild-type Cab embryos (trial 1: n = 6, trial 2: n = 2, trial 3: n = 2). Rawdata to triplicates used in Fig. 3C. IAA, hpi, hour post induction; 5-Ph-IAA (auxin analog)

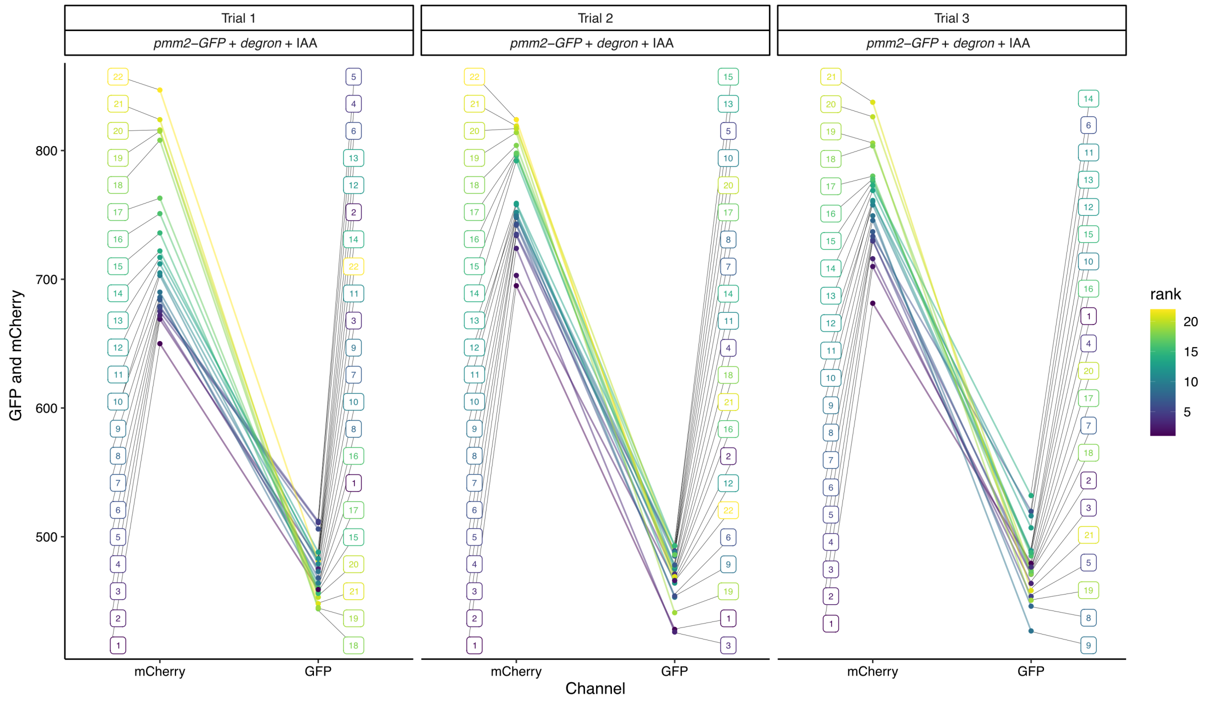

Fig. S6. No correlation between injection variability and degradation efficiency. A) End-point analysis of GFP degradation curve from Fig. 3C comparing mean GFP to corresponding mean mCherry levels in each individual (degron-injected *pmm2-GFP* embryos induced with 5 μM IAA (trial 1: n = 22, trial 2: n = 22, trial 3: n = 21)). Robust mCherry expression with low variability as tracer for the delivered degron system is matched by efficient degradation of GFP with low variation. IAA, 5-Ph-IAA (auxin analog)

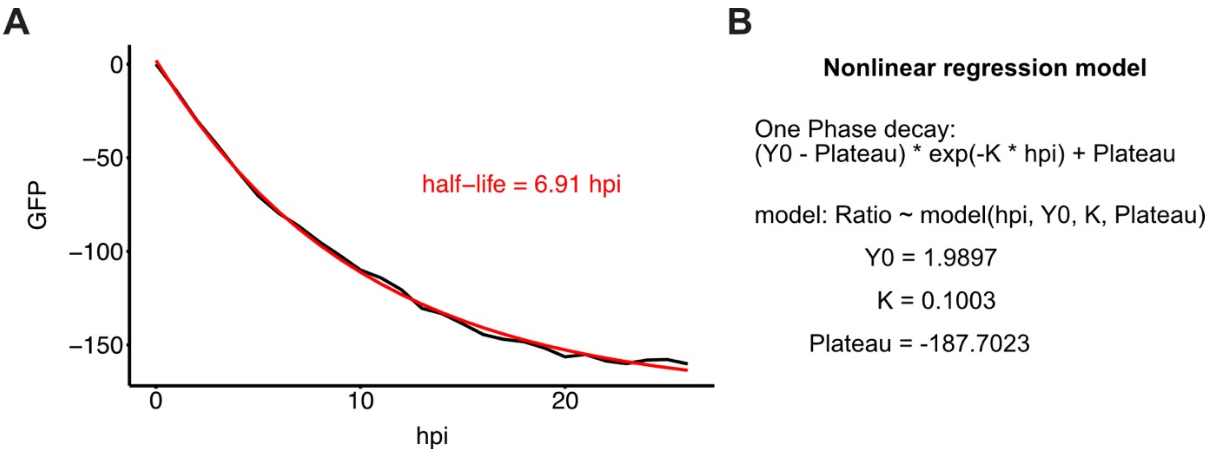

Fig. S7. One-phase exponential decay model to determine half-life of Pmm2-GFP degradation. A) Baseline corrected mean value of GFP fluorescence fitted to a one-phase exponential decay model (Y = (Y0-Plateau)exp(-k*X)+Plateau). B) Extracted values from mean GFP depletion kinetics fitted against one-phase exponential decay model. hpi, hours post induction

Biology Open • Supplementary information

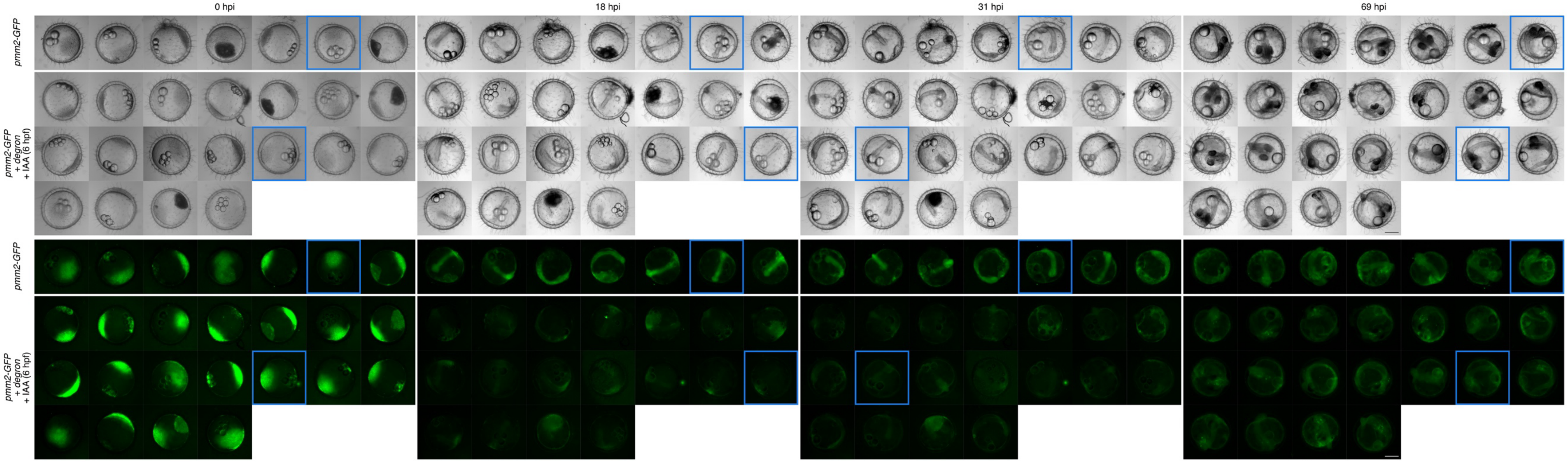

Fig. S8. No macroscopic phenotypes in GFP depleted *pmm2-GFP* and control embryos in long-term acquisition. A) Still frames from a 143 hours long acquisition of embryonic development in homozygous *pmm2-GFP* uninjected control (n = 7) and degron-injected (n = 18) and induced with 5 μM IAA at 6 hpf *pmm2-GFP* embryos, images in in brightfield and GFP fluorescence channels. Note robust depletion of GFP fluorescence at 18 and 31 hpi and recovery at 69 hpi. Scale bar 500 μM. hpf, hours post fertilization; hpi, hours post induction; IAA, 5-Ph-IAA (auxin analog); representative images used in Fig. 4 indicated by blue frame.

Biology Open • Supplementary information

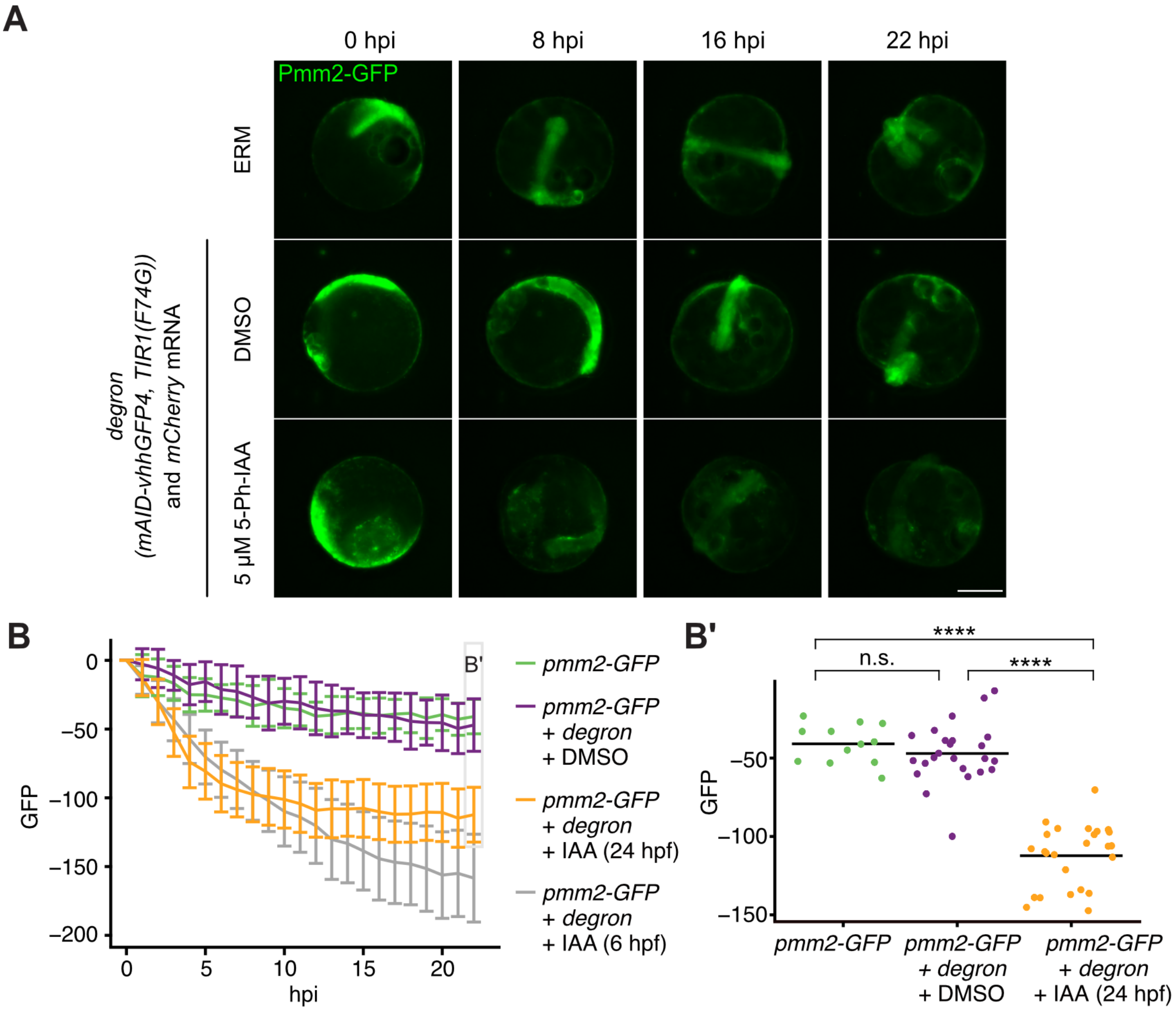

Fig. S9. Reduced degradation efficiency of Pmm2-GFP when induced at 24 hours post fertilization. A) Representative stills from time-lapse imaging of uninjected *pmm2-GFP*, degron-injected *pmm2-GFP* embryos incubated in 0.005% DMSO or 5 μM 5-Ph-IAA. Scalebar 500 μM. B) Quantification of mean GFP fluorescence following baseline correction over 22 hpi. Mean ± sd shown, uninjected *pmm2-GFP* control (n total = 12; green), *degron/mCherry* injected, non-induced embryos (n total = 24; purple), induced (n total = 25; orange). Grey line: quantification excerpt of *pmm2-GFP* degron-injected and induced at 6 hpf embryos from Fig. 3C plotted for comparison. Note less reduction of GFP (orange line) compared to earlier induction experiment (6 hpf, grey line). B') Scatterplot of raw data at 22 hpi. Mean is shown as black line, one-way ANOVA test with Tukey post-hoc multiple comparison was performed on endpoints, adjusted p-values shown, n.s. = $p > 0.05$, **** = $p \leq 0.0001$. hpf, hours post fertilization; hpi, hour post induction; IAA, 5-Ph-IAA (auxin analog)

Biology Open • Supplementary information

Biology Open (2025): doi:10.1242/bio.062081: Supplementary information

Table S1. Raw numbers of injection experiments. Raw data behind injection experiments used in Fig. 2, Fig. 3, Fig. 4 and Fig. S9. Proportion of non-injected embryos (lack of mCherry) given that were excluded from final analysis.

Injection Name	Injection Mix components	concentration	total embryos	mCherry negative	% excluded from injected	dead	analyzed
**Trial 1 - *TIR1***	*TIR1* mRNA	50 ng/μl					ERM 8
Fig. 2	*nanobody* mRNA	10 ng/μl	48	7	14.6%	8	NAA 25
	mCherry mRNA	10 ng/μl					
	non-injected		8	-	-	1	7
**Replicate 1 - *TIR1(F74G)***	*TIR1(F74G)* mRNA	50 ng/μl					DMSO 22
Fig. 3	*nanobody* mRNA	10 ng/μl	48	4	8.3%	0	IAA 22
	mCherry mRNA	10 ng/μl					
	non-injected		6	-	-	1	5
**Replicate 2 - *TIR1(F74G)***	*TIR1(F74G)* mRNA	50 ng/μl					DMSO 19
Fig. 3	*nanobody* mRNA	10 ng/μl	48	7	14.6%	0	IAA 22
	mCherry mRNA	10 ng/μl					
	non-injected		6	-	-	2	4
**Replicate 3 - *TIR1(F74G)***	*TIR1(F74G)* mRNA	50 ng/μl					DMSO 20
Fig. 3	*nanobody* mRNA	10 ng/μl	48	4	8.3%	3	IAA 21
	mCherry mRNA	10 ng/μl					
	non-injected		6	-	-	0	6
**Trial 1 - *TIR1(F74G)***	*TIR1(F74G)* mRNA	50 ng/μl					IAA 18
Fig. 4	*nanobody* mRNA	10 ng/μl	20	0	0%	2	
	mCherry mRNA	10 ng/μl					
	non-injected		10	-	-	3	7
**Trial 1 - *TIR1(F74G)***	*TIR1(F74G)* mRNA	50 ng/μl					DMSO 24
Fig. S9	*nanobody* mRNA	10 ng/μl	51	0	0%	2	IAA 25
	mCherry mRNA	10 ng/μl					
	non-injected		12	-	-	0	12

Biology Open • Supplementary information
